# The Role of microRNAs in Development of Colitis-Associated Colorectal Cancer

**DOI:** 10.3390/ijms22083967

**Published:** 2021-04-12

**Authors:** Marco Bocchetti, Maria Grazia Ferraro, Filippo Ricciardiello, Alessandro Ottaiano, Amalia Luce, Alessia Maria Cossu, Marianna Scrima, Wing-Yan Leung, Marianna Abate, Paola Stiuso, Michele Caraglia, Silvia Zappavigna, Tung On Yau

**Affiliations:** 1 Department of Precision Medicine, University of Campania “Luigi Vanvitelli”, 80131 Naples, Italy; marco.bocchetti@unicampania.it (M.B.); amalia.luce@unicampania.it (A.L.); alessiamaria.cossu@biogem.it (A.M.C.); marianna.abate@unicampania.it (M.A.); paola.stiuso@unicampania.it (P.S.); michele.caraglia@unicampania.it (M.C.); 2 Biogem Scarl, Molecular Oncology and Precision Medicine Laboratory, via Camporeale, 83031 Ariano Irpino, Italy; marianna.scrima@biogem.it; 3 Department of Pharmacy, School of Medicine and Surgery, University of Naples “Federico II”, via D. Montesano 49, 80131 Naples, Italy; mariagrazia.ferraro@unina.it; 4 Ear, Nose, and Throat Unit, AORN “Antonio Cardarelli”, 80131 Naples, Italy; filipporicciardiello@virgilio.it; 5 SSD-Innovative Therapies for Abdominal Metastases, Istituto Nazionale Tumori di Napoli, IRCCS “G. Pascale”, via M. Semmola, 80131 Naples, Italy; a.ottaiano@istitutotumori.na.it; 6 School of Science and Technology, Nottingham Trent University, Nottingham NG11 8NS, UK; 7 Division of Haematology, Department of Medicine, The University of Hong Kong, Hong Kong, China; thomas83@hku.uk; 8 John van Geest Cancer Research Centre, School of Science and Technology, Nottingham Trent University, Nottingham NG11 8NS, UK

**Keywords:** colorectal cancer, colitis-associated colorectal cancer, inflammatory bowel disease, microRNA, biomarkers

## Abstract

Colorectal cancer (CRC) is the third most deadly cancer worldwide, and inflammatory bowel disease (IBD) is one of the critical factors in CRC carcinogenesis. IBD is responsible for an unphysiological and sustained chronic inflammation environment favoring the transformation. MicroRNAs (miRNAs) belong to a class of highly conserved short single-stranded segments (18–25 nucleotides) non-coding RNA and have been extensively discussed in both CRC and IBD. However, the role of miRNAs in the development of colitis-associated CRC (CAC) is less clear. The aim of this review is to summarize the major upregulated (miR-18a, miR-19a, miR-21, miR-31, miR-155 and miR-214) and downregulated (miR-124, miR-193a-3p and miR-139-5p) miRNAs in CAC, and their roles in genes’ expression modulation in chronic colonic-inflammation-induced carcinogenesis, including programmed cell-death pathways. These miRNAs dysregulation could be applied for early CAC diagnosis, to predict therapy efficacy and for precision treatment.

## 1. Introduction

Inflammatory bowel disease (IBD) is a group of idiopathic and relapsing-remitting chronic inflammatory disorders comprising the two major subtypes: Crohn’s disease (CD) and ulcerative colitis (UC). IBDs are characterized by a susceptible genetic background, underlying immunological deregulation and intestinal microbiome dysbiosis leading to intestinal mucosa damage [1]. It is well recognized that the long-standing chronic inflammation in intestinal mucosa induces intestinal barrier injury: resulting in increased permeability and destruction of the tight junctions [2], and eventually colorectal cancer (CRC) onset [3,4]. The degree of colonic inflammation together with the disorder duration is correlated with the development of colonic neoplasia [5,6]. The influence of UC on CRC risk is approximately 2%, 8% and 18% after one, two and three decades of the disease, respectively [7]. The cumulative risk for CRC in CD is approximately 3% after 10 years, 6% after 20 years and 8% after 30 years of the disease duration [8]. Several studies have reported that UC-associated CRC has an unfavorable survival compared to sporadic CRC and is responsible for one-sixth of UC-related deaths [9,10,11].

Colitis-associated carcinogenesis is a multi-stage process starting with chronic inflammation and affected by environmental, genetic and immunologic factors, as well [12,13], eventually presenting differences when compared with sporadic CRC. In addition, drug trials in prodromal phases of IBD and/or colitis-associated CRC (CAC) appeared not completely adequate. Animal-based colitis models could improve the science capability to address exact research questions, potentially giving better disease prevention, control, and supervision. The most common CAC model is the use of dextran sulfate sodium (DSS) plus azoxymethane (AOM), other viable options are summarized in Table 1. It is important to take into account that mouse string may play a significant role in the severity and variability of the disease model and response to the same treatment.

During the cancer development, sporadic CRC (or spontaneous, unrelated to the genetics of family and CRC history) typically present a stepwise “normal mucosa-adenoma-dysplasia-carcinoma” sequence, while CAC arises as an “inflamed mucosa-dysplasia-carcinoma” sequence. Moreover, there are also unique histological and genetic alterations [22]. In clinical histopathology, CAC tissues often have a background of chronic inflammation, a higher number of signet ring cells, and a substantial portion of mucinous. Frequently, it invokes a cascade within the abnormal epithelial proliferative region, progressing to invasive adenocarcinoma from flat and non-polypoid dysplasia [9,10,12]. The difference between sporadic and CAC can also be found at the molecular level [23]. This mainly involves pro-inflammatory signaling pathways and immune responses, promoting tumorigenesis by inducing the production of inflammatory mediators, induce the expression of the anti-apoptotic genes, and stimulating cells proliferation and angiogenesis.

These processes can be regulated by microRNA (miRNA). miRNA belongs to a class of highly conserved short single-stranded segments (18–25 nucleotides) non-coding RNA, which post-transcriptionally regulate protein expression inducing messenger RNA (mRNA) degradation and/or inhibit translation of target genes binding to the 3′-untranslated regions (3′-UTR) to regulate gene expression [24,25]. During the occurrence of IBD, miRNAs play important roles either inhibiting or enhancing immune and inflammation signals by regulating the expression of the positive or negative components of immune signaling pathways associated with IBD and CRC progression. Several miRNAs studies are focusing on the CAC (Table 2); hence, the aim of this review is to discuss the major finding on this topic, and the roles of miRNAs in the progression of IBD and the CAC development.

## 2. MicroRNAs Overexpression Induces Colitis-Associated Colorectal Carcinogenesis

### 2.1. MiR-17-92 Cluster

Both miR-19a and miR-18a belong to miRNA17-92 cluster. MiR-19a has been proven to be an oncomiR, which regulate cell proliferation, differentiation, apoptosis and angiogenesis during the cancer development. In CRC, miR-19a enhances cells invasion, progression and lymph node metastasis by mediating the inhibition of Transglutaminase-2 (*TG-2*) [50], T-cell intracellular antigen 1 (*TIA1*) [51] and the inflammatory cytokine tumor necrosis factor α (*TNFα*) [52]. Overexpression of miR-19a induces epithelial–mesenchymal transition (EMT) signaling in CRC cells, confirmed by N-cadherin, Vimentin, and Fibronectin levels [52]. In DSS-induced colitis mice treated with miR-19a mimic, colon tumor numbers, sizes and tumor loads are higher compared to the control group; pro-inflammatory cytokines, including IL-1β, IL-6, IL-17a, IFN-γ and TNF-α are also upregulated [27,53]. MiR-19a mimic was also administered in the AOM/DSS-induced CRC mice and induced pro-inflammatory cytokines (IL-6, TNF-α, IL-1β and IL-17a), tumor proliferation marker (Ki-67) and NF-κB signaling markers (p-P65 and COX-2) via targeting TNF-α-induced protein 3 (*TNFAIP3*) [27]. The stimulation of TNF-α induces miR-19a expression in CAC and its overexpression activates NF-κB signaling and increases TNFAIP3. The regulatory effects of miR-19a on TNFAIP3 and NF-κB were also found in clinical tissue samples [27].

MiR-18a belongs to miRNA17-92 cluster, as well. Upregulation of miR-18a downregulates Protein Inhibitor Of Activated STAT 3 (*PIAS3*) expression and activates NF-κB and STAT3 in both CAC/CRC patients and AOM/DSS-induced mice. To be more specific, in vitro studies demonstrated that PIAS3 significantly repressed the activation of NF-κB and STAT3, while the activation of NF-κB and STAT3 transcriptionally regulate miR-18a expression level. The PIAS3/NF-κB and STAT3/miR-18a autoregulatory feedback loops are involved in cell proliferation regulation [26,54]. PIAS3 overexpression or miR-18a knockdown significantly inhibited cell proliferation in the mouse CRC xenograft model. Intracolonic administration of PIAS3 lentivirus or anti-miR-18a lentivirus in AOM/DSS-induced mice led to dramatically reduced tumor sizes/numbers, whereas knockdown of PIAS3 in CAC mice significantly promoted tumor growth [26]. Higher expression of miR-18a can be detected in feces from CRC patients [55].

### 2.2. MiR-21

MiR-21 is one of the most overexpressed and well-studied miRNA in both cancers and inflammatory-related diseases. miR-21 expression patterns can be distinguished in between IBD, CRC, CAC and normal controls and appear related to CRC patients’ survival [56,57,58,59]. The upregulation of miR-21 in cancer correlates to cell migration, invasion and proliferation, and promotes miR-21-mediated transformation in somatic cells [29,30]. In IBD, the deletion of miR-21 in C57BL/6 mice results in the exacerbation in both T-cells transfer and TNBS-induced colitis models, CD4^+^CD45R^high^ T-cells from miR-21^−/−^ mice were disposed to Th1 polarization [60]. MiR-21 knockout mice which received AOM/DSS presented a reduction of neoplasms size and numbers and induced inflammatory and carcinogenic cytokines such as IL-6, IL-17A, IL-21, and IL-23 [29]. This process further reduces BCL2 and STAT3 activation, attenuated cancer cells proliferation, simultaneously increase E-cadherin and decrease β-catenin, SOX9 and Ki-67 expressions [29]. Moreover, miR-21 expression in IBD significantly upregulates CD3^+^ T-cells and negatively correlates to PDCD4 expression in UC remission patients [59]. The abovementioned miR-21 and PDCD4 correlation can be found in CAC, as well, increasing the apoptosis, and subsequently activating NF-κB [29]. Using a non-transformed mammary epithelial cell MCF-10A overexpressing v-Src, Iliopoulos et al. [30] indicated that the transient activation of v-Src is sufficient to induce transformation. The activation of STAT3 via v-Src enhances the transcription of *MIR21*, leading to increase NF-κB and IL-6 production and inhibit PTEN.

### 2.3. MiR-31-5p

MiR-31 has both oncogenic and suppressive roles in different types of cancers. The phenotype caused by aberrant miR-31 expression seems to be strongly dependent on the endogenous expression levels. In CRC, high level of miR-31 correlates with serrated CRC [61], *KRAS* [62] and *BRAF* [61,63] mutations. MiR-31-5p activates *RAS* signaling pathway via inhibition of RASA1 translation, increasing CRC cell growth, stimulating tumorigenesis [64]. The expression of EZH2 reported as a prognostic biomarker candidate for anti-EGFR treatment [65] correlates with miR-31 serrated pathway [66,67]. Thereby, in addition to RAS signaling related genes [68], miR-31-5p could potentially be an additional predictor for precision anti-EGFR therapy [61,62,63,69]. The transcription of *MIR31* can also be activated by NF-κB and STAT3 confirmed by using LoVo CRC cells and organoids derived from mouse colon cells in response to TNF and IL-6 [36]. Moreover, miR-31 negatively correlated with *HIF1AN* expression in CRC tissue samples and cell lines compared with the corresponding adjacent normal tissue [70], and directly regulate *HIF1AN* expression in CRC confirmed by luciferase reporter assay. The presents of *HIF1AN* inhibits hypoxia-inducible factor 1α (HIF1α), and downregulation of *HIF1AN* promotes tumor angiogenesis, cell invasion and proliferation.

The induced expression of miR-31 can be identified in both UC and CD patients [35,37]. Inflamed UC mucosae showed decreased *IL13RA1* mRNA and protein expressions compared to healthy controls. MiR-31 mimics transfection in HT-29 colon cancer cells reduces both IL13RA1 protein and mRNA expression, blocks pSTAT6, *SOCS1* and *CCL26* expression. The use of miR-31 mimic also downregulate *IL13RA1* in ex vivo human inflamed UC biopsies [37]. 

MiR-31 expression also targets and inversely correlates IL-25, regulates IL-12/23-mediated Th1/Th17 inflammatory responses during the chronic inflammation process in TNBS-induced and IL-10 knockout colitis mice models [35]. IL-25 is a regulatory cytokine that has a key role in mucosal immune tolerance during inflammation response. *MIR31*-knockout DSS and TNBS-treated mice established severe colitis, induced immune responses with higher inflammatory cytokine receptors (IL7R and IL17RA) and signaling proteins (GP130) compared to the control group [36]. *IL7R*, *IL17RA* and *IL16ST* are the targets of miR-31-5p confirmed by 3ʹUTR-Luciferase reporter assays [36]. MiR-31 also regulates Hippo and WNT signaling pathways to promote epithelial regeneration [36]. The expression of miR-31 has been found dysregulated in IBD-related neoplasia compared to adjacent normal tissue in human [32,49] and the AOM/DSS-induced CRC mice models [33]. Mice with colon epithelium-specific deletion of miR-31 were utilized and presented a severe CAC compared to the wild-type mice. WD Repeat Domain 5 (WDR5) [33] and HIF1 [32] could be the targets of miR-31 for CAC development [71].

### 2.4. MiR-155

MiR-155 is a multi-functional miRNA with inflammation-related and oncogenic roles. High-level of miR-155 can be detected in CRC, promoting cells proliferation, invasion, migration, and closely related to tumor location, TNM staging, metastasis and prognosis [72,73,74,75]. MiR-155 reported as a part of a negative feedback loop controlling IL-1β and inflammatory cytokines production during LPS-mediated dendritic cells (DC) activation. This process is directly targeting TAB2, a signaling transduction adaptor of the TLR/IL-1 biochemical cascade in response to microbial stimuli [76]. miR-155 is also characterized as a macrophage response factor and affects several immune-related mediators (TLR-3, IFN-β and TNF-α) [77]. It increases IL-8 throughout the inflammatory process [78,79], modulates the inflammatory phenotype of intestinal fibroblasts and myofibroblasts via NF-κB [80]. A recent study showed that miR-155 mediates intestinal barrier dysfunction in DSS-induced mice colitis through HIF1α/TFF-3 axis [81]. The knock-down of miR-155 protects the experimental colitis mice by decreasing IFNγ, TNFα, IL-6, IL-12 and IL-17 production, reducing Th1 response and suppressing the T-cells activation by DCs [82].

MiR-155 is significantly overexpressed in affected colonic mucosa and neoplastic tissues from IBD patients compared with non-IBD controls and can be detected from the distant non-neoplastic mucosa [57]. It is also highly expressed in the tumor region from patients with CAC compared to the non-tumor colon tissues. The deletion of miR-155 in AOM/DSS-induced CRC mouse models produced a higher grade of epithelial dysplasia, number of polyps and symptom severity scores; the models also have an unfavorable survival rate compared with the control group. The enhanced tumorigenic response in miR-155 knockout mice is associated with increased neutrophils and decreased macrophages activity and to the activation of the TGFβ/SMAD signaling activity [38]. MiR-155 also in have the same effect as miR-31 [37]. The use of miR-155 mimics in both HT-29 CRC cells and in ex vivo human inflamed UC biopsies reduced mRNA *IL13RA1* expression, reduced IL-13-dependent pSTAT6 via Janus kinases (JAK) and *CCL26* and *SOCS1* expression [37].

### 2.5. MiRNA-214

MiR-214 has been reported to be a tumor-suppressor miRNA in CRC, which reduce tumor cell growth, migration, invasion [83], and modulates autophagy [84]. Downregulation of miR-214 might happen due to promoter hypermethylation [85] and correlate with liver [86,87,88], lymphatic [85], and lung metastasis in CRC patients [87]. For instance, several studies have indicated that the increased level of FGFR1 may contribute to increasing CRC liver metastasis regulated by miR-214 [86,88]. The expression of miR-214 may serve as a potential marker to predict CRC patient survival [86].

**Figure 1:**
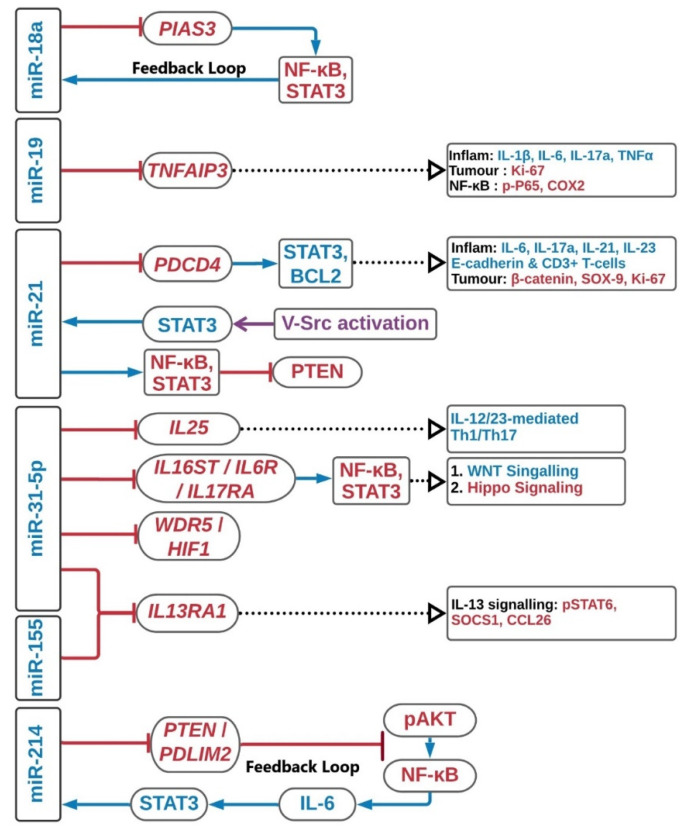
Upregulation of miRNAs in colitis-associated colorectal cancer. The schematic diagram summarizes the major pathways in the upregulation of miRNAs. Red, blue and purple in the diagram represent inhibition, regulation and enhancer, respectively. Inflam, inflammation.

In UC-associated CRC, it was reported that miR-214 was highly expressed in UC cancer compared with CD cancer and its predicted targeting action on PTEN altered the p53 signaling pathway and caused UC-induced CRC [89]. To further investigated this phenomenon, Polytarchou et al. revealed that high level of miR-214 was detected in active UC or CAC patients’ tissues, and correlated with the disease progression [39]. This correlation cannot be found in CD, IBS or healthy controls. To be more specific, miR-214 targets PDLIM2 and PTEN in UC, and suppresses NF-κB expression and AKT phosphorylation. Then, NF-κB modulates IL-6 expression which regulates STAT3 activity. STAT3-driven transcriptional activation of miR-214 triggers the positive feedback loop circuit, and the circuit is weakened in the inactive state UC. In long-standing UC, overexpression of miR-214 and hyper-activation of the inflammatory feedback loop circuit further increases the colitis-induced CRC development [39]. This feedback circuit can be blocked by a miR-214 chemical inhibitor, which reduced the size and the number of tumors and the colitis severity in several in vivo and in vitro models. The upregulated miRNAs interactions described above are summarized in Figure 1.

## 3. MicroRNAs as Suppressors of Colitis-Associated Colorectal Carcinogenesis

### 3.1. MiR-124

MiR-124 has been reported to inhibit cell proliferation and metastasis, induce apoptosis and oxidative stress, and correlate with favorable overall CRC patient survival [90,91,92]. The tumor suppression mediated by miR-124 may contribute to the maintenance of the Warburg effect [91,93], exhibiting a metabolic phenotype characterized by increased glycolysis, regardless of oxygen availability [94]. This effect is partly achieved via controlling the alternative splicing of pyruvate kinase muscle (PKM) isoforms expressions - PKM1 and PKM2 in feedback loops [95]. The expression of miR-124 targets polypyrimidine tract-binding protein 1 (PTB1) to activate the PKM1 and suppress the PKM2, which further downregulates c-Myc, E2F1 as well as STAT3 [91,96,97]. MiR-124 also activates the mitochondrial apoptosis pathway through PKM1 to facilitate HNF4α binding to the miR-124 promoter region [98] and activate oxidative stress HIF1α via PKM2 [91,99,100]. Thus, miR-124, PTB1, PKM1 and PKM2 constitute a feedback cascade and regulate cancer cells growth in human CRC [101]. MiR-124 also directly targets DDX6, which first regulates c-Myc, and further regulate the PTB1 levels [93].

In IBD, IL-6/STAT3 signaling has been reported as an important regulator in colon inflammation [102,103] in UC development and CAC progression [104] and can be modulated by miR-124. In particular, the expression of miR-124 was found inversely correlate with STAT3 in both DSS-induced and IL-10 knockout colitis mice models [43] and CRC patients, as well [105]. Downregulation of miR-124 in UC active paediatric patients resulted in upregulation of STAT3 and modulation of its related downstream targets—VEGF, BCL2, BCLXL and MMP9 [43]. Suppression of miR-124 in tissues from UC active paediatric patients was attributed to hypermethylation of STAT3 promoter region. This hypermethylation can also be found in CRC [106,107] and restored by using 5-AZA—a DNA methyltransferases inhibitor [43]. Applying nicotine as a treatment agent could ameliorate UC symptoms through upregulation of miR-124 expression by blocking STAT3 activation in DSS-induced colitis mice and epithelial cells. Therefore, targeting miR-124 and STAT3 by using nicotine may present a novel approach for treating UC [44].

### 3.2. MiR-139-5p

The role of miR-139-5p has been well studied in CRC; it is related to apoptosis, cell cycle arrest, cellular migration and invasion by targeting various coding genes [108,109,110]. Briefly, lower expression of miR-139-5p in CRC induces apoptosis, concomitantly with upregulation of apoptosis-related genes such as caspase-3, caspase-7, caspase-8 and PARP [108]; increases p27^Kip1^ and p21^Cip1/Waf1^, and also increases the G0/G1 phase regulators which cause cell cycle arrest [108]; and suppresses cell migration and invasiveness through IGF-IR/MEK/ERK signaling pathway by targeting IGF-IR, MMP-2, MMP-7 and MMP-9 [108,110]. Moreover, miR-139-5p expression can be regulated by NOTCH1, the knock-down of NOTCH1 phenocopied the inhibitory effect of miR-139-5p on CRC metastasis [108,109], inhibited EMT and enhanced the chemotherapeutic sensitivity of CRC by downregulating BCL2 [111,112]. Moreover, Bian et al. [113] reported that LINC00152—a long non-coding RNA enables to regulate NOTCH1 expression via sponging miR-139-5p, which may control CRC progression and development. 

In miR-139-5p knockout mice models, worse clinical symptoms were observed for the DSS-induced colitis compared with the wild-type mice [45,46]. The enhanced formation of intestinal neoplasia was found for the AOM/DSS-induced CRC model. The miR-139-5p knockout mice enhanced the colon inflammation and tumor development via targeting both NF-κB and Rap1b, affect NF-κB, MAPK and STAT3 signaling activities [45]. The suppression of miR-139-5p was also involving Wnt signaling, associated with cell proliferation and differentiation and promoting β-catenin nuclear accumulation [46]. Overexpression of miR-139-5p in CRC cell lines inhibited PI3K/AKT/Wnt signaling pathways through IGF-1R [46]. These results pointed out that miR-139-5p act as a protective agent against both colitis and CRC, maintaining intestinal homeostasis.

### 3.3. MiR-193a-3p

**Figure 2:**
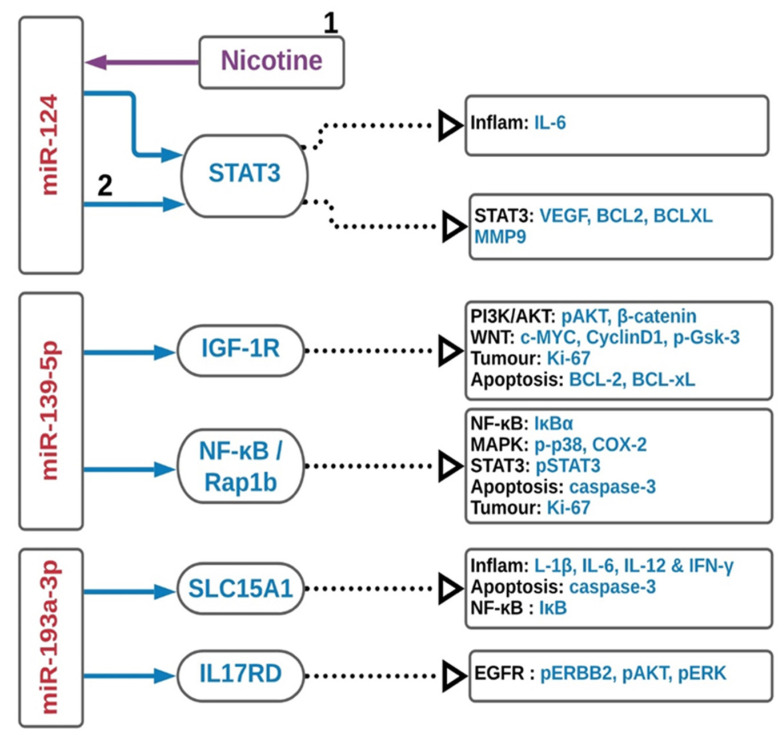
Downregulation of miRNAs in colitis-associated colorectal cancer. The schematic diagram summarizes the major pathways in the downregulation of miRNAs. Red, blue and purple in the diagram represent inhibition, regulation and enhancer, respectively. Inflam, inflammation.

Low expression of miR-193a-3p in CRC is associated with an unfavorable prognosis [114,115]: This miRNA acts as a tumor suppressor miRNA targeting *KRAS* and correlates with *BRAF*-mutated CRC [116]. The expression of miR-193-3p is significantly downregulated in UC active, UC-induced neoplasia and UC-driven CRC compared to adjacent normal tissues [48,49]. Studies revealed that miR-193-3p expression inversely correlates with *SLC15A1*, which is a positive TLR4-mediated inflammatory response regulator, and subsequently suppress NF-κB signaling. The use of miR-193a-3p mimic in CRC tissue significantly relief the colitis symptoms in DSS-induced colitis mice model. Overexpression of SLC15A1 by using SLC15A1 3′-UTR mutant lentiviral vector neutralized the anti-inflammatory effect of miR-193a-3p [48]. IL17RD in UC-associated CRC directly targets and inversely correlates with miR-193a-3p. MiR-193a-3p transfection reduced cells proliferation acting on EGFR signaling by targeting IL17RD; moreover, it regulates pAKT, pERK and pERBB2 expression [49]. The downregulated miRNAs interactions described above are summarized in Figure 2. 

## 4. MicroRNAs as a Treatment Tool for Inflammatory Bowel Disease

The advantage of miRNA-based therapy is to differentially modulate target genes at post-transcriptional levels in multi-pathological pathways and appeared to be more flexible over siRNAs, negatively regulating target genes [117]. MiRNA-based therapies comprise two fundamental strategies: miRNA mimicry and antagonism [118]. Using miRNA mimics to restore miRNA expression have been extensively applied in miRNA research, including miR19a [27], miR-193a-3p [48], miR-31 [37] and miR-155 [37] studies mentioned above. Mimic particles can also be encapsulated into oxidized konjac glucomannan (OKGM) microspheres [36]. For example, the administration of peptosome-MIR31 via enema into the large intestines of mice with DSS-induced colitis demonstrated a reduction of inflammatory response, gain in body weight and increased colon length, promoting epithelial cell proliferation [36]. On the other hand, it is possible to reduce miRNA overexpression aberrantly acting on its target genes by the use of miRNA antagonists [119]; for example, Lu et al. [120] reported that the antagomir miR-155 alleviated DSS-induced colitis in mice by targeting *SHIP-1* and noted that individual mRNA may be regulated by more than one miRNA, and each miRNA may regulate numerous mRNAs [121]. Moreover, the existence of long non-coding RNAs giving another layer of complexity, forming a complex biological regulation, leading to unexpected outcomes [122]. Thus, miRNA delivery is a vital challenge. Since miRNAs might act on different mRNA targets, with different abundance from tissue to tissue, possibly leading to undesired effects, the targeted delivery strategy is crucial for two reasons: first to prioritize target tissue and avoid unspecific and random tissue distribution and also to stabilize and preserve miRNAs structure and properties in physiological fluids [123].

## 5. MicroRNAs as Predictive Inflammatory Bowel Disease Biomarker(s)

The IBD treatment goal is to obtain remission and recovery of the altered mucosa, in order to avoid or sizing surgical intervention. It is important to mention that the latest therapies target inflammatory processes, for example, pro-inflammatory cytokine TNF inhibitors. However, approximately 30% of the patients are not responding to the treatment from the beginning (primary non-responders), while 50% of the primary responders start to lose the initial therapeutic benefit over time (secondary non-responders) [124,125,126]. Morilla et al. [127] reported that the neural-network-developed algorithms utilized a total of nine miRNAs with five clinical features in IBD patients associated with anti-TNF monoclonal antibody therapy treatment response. Moreover, fecal-based miRNA screening has been investigated to find the pattern to detect colon diseases, including CRC [55,56,128,129,130] and IBD [131,132], in the early phases with non-invasive procedures. For example, stool miR-16, miR-21, miR-223 and miR-1246 expression resulted in being upregulated in active CD and UC patients compared to healthy controls and circulating miRNAs detection may further prevent the chance of *Clostridioides difficile* infection [133] in IBD patients. Thus, miRNAs are the potential biomarkers to further investigate for an early non-invasive IBD screening [134]. 

## 6. Conclusions

MiRNAs play a variety of biological roles in cancer and (chronic) inflammation development, which induces an unphysiological condition favoring the malignant transformation. MiRNAs nowadays are becoming more and more important because of their role in biochemical pathways regulation, and some of them are showing promising therapeutic effects. There are a wide variety of genes finely regulated by miRNAs; the strategy, as we mentioned, could be utilized to restore their physiological levels to maintain the homeostasis. It is also important to take into account that miRNAs may have different binding sites on different targets, and their deregulation is not easy to control, which is also facing the similar challenge to the conventional pharmacological drugs development. To build a real translational therapeutic approach, it is important to study the physiological levels, first in silico and then in vitro and in vivo to compare with healthy and cancerous tissues. The next step will be to clarify and assess the putative mechanism of action and the corresponding target genes involved in miRNA regulation and the effectiveness of the pathology. Moreover, miRNAs may be exploited as biomarkers; their differential expression is important for early diagnosis and even to predict CRC conventional chemotherapy response. We hope this manuscript will help future speculation and discussion on CRC diagnosis and precision treatments, with the involvement of those promising small non-coding RNAs.

## Figures and Tables

**Table 1:** The most commonly used animal models in colitis and colitis-associated colorectal tumorigenesis.

Type	Method	Prevalent of Response	Limitations
Colitis Models			
Chemically Induced	DSS	Epithelial damage	Does not require T and/or B cell responses [14], high severity variability [15]
	TNBS/DNBS	Epithelial damage, Immune-driven	Aetiopathogenesis not clear [16]
	Oxazolone	Epithelial damage, Immune-driven	International administration required [15]
Spontaneous Mutation	SAMP1/Yit	Immune-driven	Affect small intestine only [17], low breeding rate [15]
	C3H/HeJBir	Immune-driven	Greatly influenced by caging conditions [15]
Adoptive T Cell Transfer	CD4^+^CD45RB^hi^	Immune-driven	Lack of a full overview of colitis development [18], expensive
Genetically Engineered	IL-10^−/−^	Immune-driven	Lack of focal granulomatous inflammation and Transmural inflammation [17]
Colitis-Associated Colorectal Tumorigenesis Models			
Chemically Induced	DSS	Epithelial damage	Low cancer incidents [19].
	AOM/DSS	Epithelial damage	The most common CAC model [20]
Genetically Engineered	IL-10^−/−^	Immune-driven	~60% of cancer Incidence [21]

AOM, Azoxymethane; DSS, dextran sulfate sodium; TNBS, 2,4,6-trinitrobenzene sulfonic acid; DNBS, dinitrobenzene sulfonic acid.

**Table 2:** Major microRNA studies in colitis-associated colorectal cancer.

miRNA	Target Gene(s)	Function	Reference(s)
Upregulation			
miR-18a	*PIAS3*	Proliferation, cell apoptosis	[26]
miR-19a	*TNFAIP3*	Activate NF-κB signaling	[27]
miR-21	*PDCD4*, *PTEN*	Invasion, intravasation, metastasis, apoptosis	[28,29,30]
miR-26b	*CCNDBP1*	Tumorigenesis and development of digestive diseases	[31]
miR-31	*HIF1*, *WDR5*, *IL13RA1*	Activate RAS signaling, stimulating tumorigenesis and correlates with serrated CRC	[32,33,34,35,36]
miR-146b	*TRAF6*, *IRAK1*		[34]
miR-155	*IL13RA1*	Negative feedback loop controlling IL-1β	[34,37,38]
miR-181b-1	*CYLD*	Cellular transformation	[30,34]
miR-214	*PDLIM2*, *PTEN*	Malignant transformation	[39]
miR-221	*PDLIM2*		[34]
miR-223	*RASA1*	Cell proliferation	[40]
miR-301a	*BTG1*	Promote intestinal inflammation	[41]
Downregulation			
miR-34a	*IL6/EMT/EGR1*	Suppresses migration and invasion	[34,42]
miR-124	*STAT3/ROCK1*	Inhibits neoplastic transformation	[43,44]
miR-139-5p	*IGF-1R*	Maintain intestinal homeostasis	[45,46]
miR-185-3p	*MLCK*	Regulate via lncRNA *CCAT1*	[47]
miR-193a-3p	*SLC15A1*	Suppress NF-κB signaling	[48,49]

CRC, colorectal cancer; lncRNA, long non-coding RNA.

## Abbreviations

BTG1	BTG Anti-Proliferation Factor 1
CCNDBP1	Cyclin D1 Binding Protein 1
CYLD	CYLD Lysine 63 Deubiquitinase
EGR1	Early growth response protein 1
EMT	Epithelial–mesenchymal transition
HIF1	Hypoxia-inducible factor 1
IGF-1R	Insulin-like growth factor 1
IL13RA1	Interleukin 13 Receptor Subunit Alpha 1
IL6	Interleukin-6
IRAK1	Interleukin-1 receptor-associated kinase 1
MLCK	Myosin light chain kinase
PDCD4	Programmed Cell Death 4
PDLIM2	PDZ And LIM Domain 2
PIAS3	Protein Inhibitor Of Activated STAT 3
PTEN	Phosphatase and tensin homolog
RASA1	RAS p21 protein activator 1
ROCK1	Rho Associated Coiled-Coil Containing Protein Kinase 1
SLC15A1	Solute Carrier Family 15 Member 1
STAT3	Signal transducer and activator of transcription 3
TNFAIP3	TNF alpha induced protein 3
TRAF6	TNF Receptor Associated Factor 6
WDR5	WD Repeat Domain 5
